# Supplementary material for: Crawling and Gliding: A Computational Model for Shape-Driven Cell Migration
Source: PLoS Comput Biol. 2015 Oct 21;11(10):e1004280. doi: 10.1371/journal.pcbi.1004280 (PMC4619082; doi:10.1371/journal.pcbi.1004280)
Supplement: S1 Code — (ZIP) [file pcbi.1004280.s012.zip › release/tst/doc/html/info_8h_source.html]

Tissue Simulation Toolkit: info.h Source File


Tissue Simulation Toolkit
 0.1.4.1


- Main Page
- Namespaces
- Classes
- Files

- File List
- File Members

info.h

Go to the documentation of this file.

1 /\*

2

3 Copyright 1996-2006 Roeland Merks

4

5 This file is part of Tissue Simulation Toolkit.

6

7 Tissue Simulation Toolkit is free software; you can redistribute

8 it and/or modify it under the terms of the GNU General Public

9 License as published by the Free Software Foundation; either

10 version 2 of the License, or (at your option) any later version.

11

12 Tissue Simulation Toolkit is distributed in the hope that it will

13 be useful, but WITHOUT ANY WARRANTY; without even the implied

14 warranty of MERCHANTABILITY or FITNESS FOR A PARTICULAR PURPOSE.

15 See the GNU General Public License for more details.

16

17 You should have received a copy of the GNU General Public License

18 along with Tissue Simulation Toolkit; if not, write to the Free

19 Software Foundation, Inc., 51 Franklin St, Fifth Floor, Boston, MA

20 02110-1301 USA

21

22 \*/

23

32 class Graphics;

33 class Dish;

34 //class ostream;

35

36 class Info {

37

38 public:

39

46  Info(Dish &dish, Graphics &graphics, std::ostream &out=std::cout);

47

54  void Menu(void);

55

58  void WriteCOM(int cell\_id, std::ostream &out=std::cout);

59

62  Cell &ClickCell(Graphics \*g);

63

64 private:

65  Info(void);

66

67

68 private:

69  Graphics \*graphics;

70  Dish \*dish;

71  std::ostream \*os;

72 };

Cell

**Definition:** cell.h:33

Info::ClickCell

Cell & ClickCell(Graphics \*g)

Waits until the user clicks a cell and returns a reference to it.

**Definition:** info.cpp:220

Dish

The virtual Petri dish.

**Definition:** dish.h:43

Graphics

API for Graphics windows.

**Definition:** graph.h:36

Info::WriteCOM

void WriteCOM(int cell\_id, std::ostream &out=std::cout)

Writes center of mass of cell "cell\_id" to stream out.

**Definition:** info.cpp:228

Info::Menu

void Menu(void)

Reads out key presses in the Graphics window and interprets them.

**Definition:** info.cpp:52

Info

Enables interactive querying of the simulation.

**Definition:** info.h:36


---

Generated on Thu Aug 14 2014 22:04:01 for Tissue Simulation Toolkit by  

 1.8.6
